# Differences in the transcriptional immune response to *Albugo candida* between white rust resistant and susceptible cultivars in *Brassica rapa* L.

**DOI:** 10.1038/s41598-023-35205-5

**Published:** 2023-05-26

**Authors:** Naomi Miyaji, Mst. Arjina Akter, Motoki Shimizu, Hasan Mehraj, Md Asad-Ud Doullah, Elizabeth S. Dennis, Izumi Chuma, Ryo Fujimoto

**Affiliations:** 1 Graduate School of Agricultural Science, Kobe University, Kobe, 657-8501 Japan; 2 Iwate Biotechnology Research Center, Narita, Kitakami, Iwate 024-0003 Japan; 3 Department of Plant Pathology, Faculty of Agriculture, Bangladesh Agricultural University, Mymensingh, 2202 Bangladesh; 4 Department of Plant Pathology and Seed Science, Faculty of Agriculture, Sylhet Agricultural University, Sylhet, 3100 Bangladesh; 5 CSIRO Agriculture and Food, Canberra, ACT 2601 Australia; 6 School of Life Science, Faculty of Science, University of Technology Sydney, Broadway, NSW 2007 Australia; 7 Obihiro University of Agriculture and Veterinary Medicine, Obihiro, 080-8555 Japan

**Keywords:** Plant sciences, Plant immunity, Plant molecular biology, Plant signalling, Plant stress responses

## Abstract

*Albugo candida* causing white rust disease decreases the yield of *Brassica rapa* vegetables greatly. Resistant and susceptible cultivars in *B. rapa* vegetables have different immune responses against *A. candida* inoculation, however, the mechanism of how host plants respond to *A. candida* is still unknown. Using RNA-sequencing, we identified differentially expressed genes (DEGs) between *A. candida* inoculated [48 and 72 h after inoculation (HAI)] and non-inoculated samples in resistant and susceptible cultivars of komatsuna (*B. rapa* var. *perviridis*). Functional DEGs differed between the resistant and susceptible cultivars in *A. candida* inoculated samples. Salicylic acid (SA) responsive genes tended to be changed in their expression levels by *A. candida* inoculation in both resistant and susceptible cultivars, but different genes were identified in the two cultivars. SA-dependent systemic acquired resistance (SAR) involving genes were upregulated following *A. candida* inoculation in the resistant cultivar. Particular genes categorized as SAR that changed expression levels overlapped between *A. candida* and *Fusarium oxysporum* f. sp. *conglutinans* inoculated samples in resistant cultivar, suggesting a role for SAR in defense response to both pathogens particularly in the effector-triggered immunity downstream pathway. These findings will be useful for understanding white rust resistance mechanisms in *B. rapa.*

## Introduction

The genus *Brassica* provides vegetables, oilseeds, condiments, and fodder crops that are significant sources of nutrition and health-promoting substances such as vitamins, minerals, dietary fiber, and phytochemicals^1–3^. This genus includes *Brassica rapa* L., *Brassica oleracea* L., *Brassica napus* L., and *Brassica juncea* (L.) Czern & Coss. *B. rapa* contains leafy vegetables such as Chinese cabbage (var. *pekinensis*), pak choi (var. *chinensis*), and komatsuna (var. *perviridis*), root vegetables such as turnip (var. *rapa*), and oilseed (var. *oleifera*). *B. oleracea* provides commercially important vegetable crops with morphological variations such as cabbage (var. *capitata*), broccoli (var. *italica*), kale (var. *acephala*), kohlrabi (var. *gongylode*s), and cauliflower (var. *botrytis*)^3^. The oilseed crop, canola/rapeseed is included in *B. napus*, and in *B. juncea*, Indian mustard, brown and leaf mustards, and Sarepta mustard are included^3,4^. *B. rapa* (AA genome) is one of the ancestral species of *B. juncea* (AABB genome) and *B. napus* (AACC genome). Other ancestral species are *Brassica nigra* L.(BB genome) and *B. oleracea* (CC genome)^5^.

Most *B. rapa* vegetables are F_1_ hybrid cultivars^6^, and disease resistance is a high priority for developing new F_1_ hybrid cultivars^7,8^. Pathogens such as fungi, bacteria, and viruses reduce production in *B. rapa* vegetables^7,8^, and white rust is a major disease. White rust is caused by an obligate biotrophic oomycete pathogen, *Albugo candida*, and the symptoms of this disease appear as white to cream-colored zoosporangial pustules on cotyledons, leaves, and stems^9^. This disease spreads to the leaf surface, stem, or inflorescences, and the pustules become more prominent as the disease progresses^10^. White rust infection damages the value of products and reduces seed formation leading to significant yield losses not only in *B. rapa* vegetables but also in *B. juncea* (Indian mustard) and *B. napus* (canola/rapeseed)^2,11^.

There are two types of plant immunity, pathogen/microbe-associated molecular pattern (PAMP/MAMP)-triggered immunity (PTI) and effector-triggered immunity (ETI)^12,13^. Basically, PTI is the first barrier and stimulates defense gene expression such as the mitogen-associated protein kinase (MAPK) cascade or WRKY transcription factors to protect against pathogen invasion. Pathogens supply avirulence (AVR) molecules/proteins called effectors to suppress PTI. The failure of PTI defense helps to activate an immune response, which is called effector-triggered immunity (ETI). PTI is governed by cell surface-localized pattern recognition receptors by the recognition of PAMPs/MAMPs, and effector-triggered immunity is activated by host resistance (R) proteins. This recognition of specific effectors by R proteins is termed “gene-for-gene resistance” or “gene for gene theory”^12^. PTI is comparatively weaker than ETI against newly adapted pathogens in host plants^13^. ETI leads to hypersensitive reaction (HR) including programmed cell death, synthesis of plant hormones, and expression of defense-related genes. Recently it was reported that ETI and PTI cross talked and shared the same downstream responses^14–16^. In general, the salicylic acid (SA) signaling pathway contributes to resistance against biotrophic and hemibiotrophic pathogens, while the jasmonic acid (JA) and ethylene (ET) signaling pathways contribute to resistance against necrotrophic pathogens^17–20^. Basically, the SA pathway and the JA/ET pathway act antagonistically, but there are some reports that these pathways interact synergistically^18–24^.

RNA-sequencing (RNA-seq) gives accurate global expression profiling not only of protein-coding genes but also of noncoding RNAs. It also can be applied to detect allele-specific expression and alternative splicing variants^25–29^. RNA-seq can monitor expression of pathogen responsive genes in host plants and provide insights into the network, pathways, or genes involved in the plant immune response against the pathogen^30,31^. In *B. rapa*, the transcriptional response against *Plasmodiophora brassicae* that causes clubroot^32,33^, *Fusarium oxysporum* f. sp. *conglutinans* (*Foc*) that causes Fusarium yellows^34^, *Pectobacterium carotovorum* ssp. *carotovorum* that causes soft rot^35^, or *Hyaloperonospora brassicae* that causes downy mildew^36^ have been examined by RNA-seq and important pathways or candidate genes involved in the resistance mechanisms have been identified. SA-responsive genes have also been identified by RNA-seq and combined with the transcriptome analyses of SA treatment and *Foc* infection has shown that SA-responsive genes [i.e., genes involved in systemic acquired resistance (SAR)] induced by *Foc* infection may play an important role in the defense response to *Foc*^37^.

The aim of this study was to gain insights into the immune responses of host plants against *A. candida* infection in *B. rapa* vegetables. We performed RNA-seq at 48 and 72 h after *A. candida* inoculation in resistant and susceptible komatsuna cultivars to examine the broad disease responses. Genes differentially expressed between non-inoculated and inoculated plants and between white rust resistant and susceptible cultivars were identified. Our study will be useful for identifying pathways or genes associated with the defense response to *A. candida* and contribute to understanding the resistance mechanism.

## Materials and methods

### Plant materials and fungal materials

Two commercial F_1_ hybrid cultivars of komatsuna (*B. rapa* var. *perviridis*), ‘Nanane’ (Takii & Co., Ltd., Kyoto, Japan) and ‘Misugi’ (Sakata Seed Corporation, Yokohama, Japan), were used as plant materials. Research carried out on plant material is comply with relevant institutional, national, and international guidelines and legislation.

*Albugo candida* of Mibuna isolate WMB01 was originally isolated from Mibuna (*B. rapa* var. *lacinofolia*) in a field in Higashi-ohmi, Shiga, Japan in 2013. Another turnip isolates WKB01 was originally isolated from a turnip (*B. rapa* var. *rapa*) in a field in Kobe, Hyogo, Japan in 2018. For maintaining WMB01 and WKB01, seedlings of ‘Misugi’ were used. Seven-day-old plants were inoculated through spraying WMB01 or WKB01 with the concentration of 1 × 10^5^ zoosporangia/ml, then incubated in a moist chamber for 24 h at 22 °C under dark conditions and the plants were moved to a growth chamber and kept under growth conditions of 16 h light and 8 h dark at 21 °C, with regular irrigation. Every three to 4 weeks inoculation, the above process was repeated for maintenance. WMB01 and WKB01 were maintained at the laboratory of horticultural crop propagation, at Kobe University.

### Inoculation test

Seeds of ‘Nanane’ and ‘Misugi’ cultivars were sown on soil and kept under 16 h light and 8 h dark at 21 °C. Seven-day-old plants were inoculated through spraying WMB01 or WKB01 with the concentration of 1 × 10^5^ zoosporangia/ml. To confirm successful inoculation, plants were incubated in a dark growth chamber for 24 h at 22 °C with 100% humidity, and the plants were moved to a growth chamber and kept under growth conditions of 16 h light and 8 h dark at 21 °C, with regular irrigation. Ten days after inoculation, ‘Misugi’ showed many white-colored zoosporangial pustules on the adaxial and abaxial side of cotyledons, while ‘Nanane’ did not show any pustules on both sides of cotyledons (Supplementary Figure S1), indicating that ‘Nanane’ and ‘Misugi’ are resistant and susceptible to *A. candida*, respectively. For gene expression studies, one cotyledon of each plant was harvested after 24, 48, or 72 h inoculation of *A. candida*, and frozen in liquid nitrogen.

For gene expression study using true leaves, 14-day-old seedlings were inoculated through spraying WKB01 with the concentration of 1 × 10^5^ zoosporangia/ml. The resistance of ‘Nanane’ and the susceptibility of ‘Misugi’ were confirmed by maintaining the plants in a dark growth chamber for 24 h at 22 °C with 100% humidity, and the plants were moved to a growth chamber and kept under growth conditions of 16 h light and 8 h dark at 21 °C, with regular irrigation. The first true leaf of each seedling was harvested after 72 h inoculation of WKB01, and frozen in liquid nitrogen.

### RNA-sequencing

Total RNA from cotyledons was extracted by SV Total RNA Isolation System (Promega Co., Madison, WI, USA). Twelve sequence libraries were prepared for RNA-sequencing (RNA-seq), (1) N_0HAI, samples without *A. candida* inoculation in ‘Nanane’ (two replicates); (2) N_48HAI, samples with *A. candida* (WMB01) inoculation at 48 h after inoculation (HAI) in ‘Nanane’ (two replicates); (3) N_72HAI, samples with *A. candida* (WMB01) inoculation at 72 HAI in ‘Nanane’ (two biological replicates); (4) M_0HAI, samples without *A. candida* inoculation in ‘Misugi’ (two replicates); (5) M_48HAI, samples with *A. candida* (WMB01) inoculation at 48 HAI in ‘Misugi’ (two replicates); (6) M _72HAI, samples with *A. candida* (WMB01) inoculation at 72 HAI in ‘Misugi’ (two replicates). 1 µg of total RNA was used to prepare each sequencing library with an RNA Sample Prep Kit v. 2 (Illumina, San Diego, CA, USA).

### Differentially expressed genes (DEGs) detection and gene ontology (GO) analysis

RNA-seq was performed using Nextseq500 (75 bp read length, paired-end). Sequenced reads were quality checked by FastQC version 0.11.8 and low-quality reads were trimmed using Trimmomatic version 0.36^38^. HISAT2 version 2.1.0^39^ was used to align the trimmed reads to the *B. rapa* reference genome version 1.5 (http://brassicadb.cn). The number of clean reads and the percentage of mapped reads are shown in Supplementary Table S1. Gene expression levels [fragments per kilo-base per million (FPKM)] were scored using cuffdiff v.2.2.1^40^. Differentially expressed genes (DEGs) with and without *A. candida* inoculation were identified based on two criteria of two-fold difference (|log 2 ratio|≥ 1.0) and 95% confidence. The gene ontology (GO) tool, agriGO^41^, was used for enrichment analysis of gene functional ontology term following the methods described by Shimizu et al.^42^.

### Gene expression analysis

For validation of RNA-seq analysis by real-time RT-PCR (qPCR), we used total RNA isolated from cotyledons with and without inoculation with *A. candida* (WMB01) independently harvested from RNA-seq. Using *A. candida* isolate (WKB01), total RNA isolated from cotyledons or true leaves with and without inoculation was used. cDNA was synthesized from 500 ng total RNA using ReverTra Ace^®^ qPCR RT Master Mix with gDNA Remover (TOYOBO Co., Ltd., Osaka, Japan). The specificity of the primer set of each gene was first tested by electrophoresis of RT-PCR amplified products using QuickTaq^®^HS DyeMix (TOYOBO) on 1.5% agarose gel in which single products were observed. RT-PCR conditions were 94 °C for 2 min followed by 35 cycles of 94 °C for 30 s, 55 °C for 30 s, and 68 °C for 30 s. The absence of genomic DNA contamination was confirmed by the PCR of no RT control^43,44^. qPCR was performed using a LightCycler 96 (Roche Molecular Systems, Inc., Pleasanton, CA, USA). cDNA was amplified using FastStart Essential DNA Green Master (Roche). qPCR conditions were 95 °C for 10 min followed by 40 cycles of 95 °C for 10 s, 60 °C for 10 s, and 72 °C for 10 s, and the Melting program (65–97 °C at 0.1 °C/s)^43^. After amplification cycles, each reaction was subjected to melt temperature analysis to confirm the presence of single amplified products. The expression level of each gene relative to *ACTIN* (*Bractin*) was automatically calculated using automatic CQ calling according to the manufacturer’s instruction (Roche)^45^. Data presented are the average and standard error of three biological and technical replicates and statistically analyzed using Dunnett’s test or Student’s *t* test, *p* value < 0.05 or 0.01. The primer sets are listed in Supplementary Table S2.

## Results

### Determination of time points for detecting differentially expressed genes following *A. candida* inoculation

**Figure 1:**
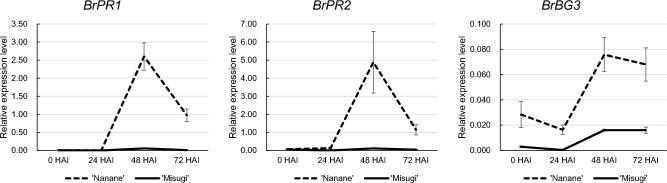
Gene expression levels after *A. candida* (WMB01) inoculation. Expression levels of three genes were measured by real-time RT-PCR at 24, 48, and 72 h after *A. candida* inoculation (HAI). Values are mean ± SE (three biological and technical replicates) for relative expression levels compared with *Bractin*.

The komatsuna white rust resistant cultivar ‘Nanane’ and susceptible cultivar ‘Misugi’ were used. To determine the time points for detecting differentially expressed genes (DEGs) following *A. candida* inoculation, we inoculated 7-day seedlings WMB01 and harvested cotyledons at 0, 24, 48, and 72 h after *A. candida* inoculation (HAI). The phytohormone SA contributes to the activation of the defense response against biotrophic pathogens such as *A. candida*^17–20^, so the transcriptional response following *A. candida* inoculation in three SA-induced genes, *PATHOGENESIS-RELATED 1* (*BrPR1*), *BrPR2*, and *BETA-1*,*3-GLUCANASE 3* (*BrBG3*)^37^, was examined. In the resistant cultivar, ‘Nanane’, the expression levels of *BrPR1*, *BrPR2*, and *BrBG3* were highest at 48 HAI, then decreased at 72 HAI, but the expression levels at 72 HAI were higher than at 0 or 24 HAI (Fig. 1). In contrast, the susceptible cultivar, ‘Misugi’, showed a low level of expression of the three genes at all times following *A. candida* inoculation (Fig. 1). These results indicate that 48 and 72 HAI are suitable times for detecting the differences in transcription between resistant and susceptible cultivars following *A. candida* inoculation.

### Identification of differentially expressed genes following *A. candida* inoculation

**Figure 2:**
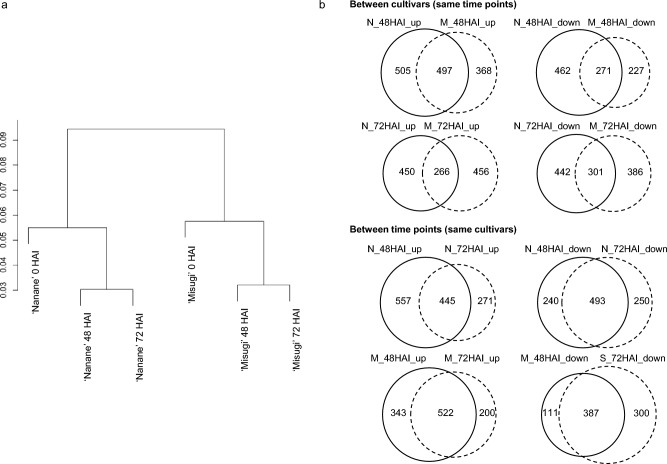
Comparison of differentially expressed genes at 48 and 72 h after *A. candida* (WMB01) inoculation (HAI) in resistant (‘Nanane’, N) and susceptible (‘Misugi’, M) cultivars. (**a**) Clustering analysis using fragments per kilobase per million (FPKM) values of all genes. (**b**) Venn diagram showing the number of up or downregulated genes between cultivars or between time points.

RNA-seq was performed at 0, 48, and 72 HAI in ‘Nanane’ and ‘Misugi’. From 8.3 to 11.3 M reads were mapped to the reference genome, and from 5.8 to 9.2 M reads (about 70–80% of total reads) were uniquely mapped to the reference genome (Supplementary Table S1). DEGs between with and without *A. candida* (WMB01) inoculation were identified using the following criteria, the two-fold difference (|log2| ratio ≥ 1) and 95% FDR. At 48 HAI, 1735 DEGs including 1002 upregulated (N_48HAI_up) and 733 downregulated genes (N_48HAI _down) were identified in ‘Nanane’. In ‘Misugi’, 1363 DEGs including 865 upregulated (M_48HAI _up) and 498 downregulated DEGs (M_48HAI _down) were identified (Table 1). At 72 HAI, 1459 DEGs including 716 upregulated (N_72HAI _up) and 743 downregulated genes (N_72HAI _down) were identified in ‘Nanane’, and in ‘Misugi’, 1409 DEGs including 722 upregulated (M_72HAI _up) and 687 downregulated DEGs (M_72HAI _down) were identified (Table 1). Hierarchical clustering analysis revealed that the two cultivars were separated; 48 HAI and 72 HAI samples were clustered (Fig. 2a). More genes overlapped between two time points in the same cultivar (44.4–77.7%) than between the two cultivars at the same time point (36.8–57.5%) (Fig. 2b, Table 1).

**Table 1:** Number of differentially expressed genes between with and without *A. candida* (WMB01) inoculation.

*A. candida*		SA			*F. oxysporum*		
		Up	Down	Total	Up	Down	Total
‘Nanane’							
48 HAI							
Up	1002	146	118	264	47	0	47
Down	733	101	207	308	7	1	8
Total	1735	247	325	572	54	1	55
72 HAI							
Up	716	146	119	265	25	0	25
Down	743	89	195	284	6	1	7
Total	1459	235	314	549	31	1	32
‘Misugi’							
48 HAI							
Up	865	93	170	263	18	0	18
Down	498	127	73	200	7	1	8
Total	1363	220	243	463	25	1	26
72 HAI							
Up	722	122	126	248	26	0	26
Down	687	160	110	270	7	1	8
Total	1409	282	236	518	33	1	34
‘Nanane’& ‘Misugi’							
48 HAI							
Up	497	18	55	73	1	0	1
Down	271	52	29	81	3	1	4
Total	768	70	84	154	4	1	5
72 HAI							
Up	266	27	33	60	4	0	4
Down	301	42	32	74	3	1	4
Total	567	69	65	134	7	1	8

*SA* number of overlapped genes with up or downregulated genes at 72 h after salicylic acid treatments^37^.

*F. oxysporum*, number of overlapped genes with up or downregulated genes after 24 h *Fusarium oxysporum* f. sp. *conglutinance* inoculation in the resistant line, RJKB-T23^34^.

Six genes, *BrPR1*, *BrPR2*, *BrBG3*, *ADENOSINE-5'-PHOSPHOSULFATE KINASE 1* (*BrAPK1*), *GLUTATHIONE S-TRANSFERASE F9* (*BrGSTF9*), and *SUPERROOT 1* (*BrSUR1*), were selected for validation of RNA-seq analysis by qPCR. The expression pattern determined by qPCR corresponded to the RNA-seq patterns in all six genes of both cultivars, except for the expression of *BrAPK1* in ‘Nanane’ (Supplementary Figure S2).

The expression pattern of six genes following inoculation by a different isolate, WKB01, was examined by qPCR. Seven-day seedlings were inoculated with WKB01 and cotyledons were harvested at 0 and 72 HAI. The expression of all six genes was induced by WKB01 inoculation in both cultivars, except for *BrAPK1* and *BrSUR1* in ‘Nanane’ (Supplementary Figure S3). The expression pattern of all genes following WKB01 inoculation corresponds to that following WMB01 inoculation (Supplementary Figures S2, S3). The expression pattern of six genes in true leaves was also examined. Fourteen-days seedlings were inoculated with WKB01 and the first leaves were harvested at 0 and 72 HAI. The expression of all six genes was induced by WKB01 inoculation in both cultivars (Supplementary Figure S3). The expression pattern of all genes in true leaves corresponded to that of cotyledons except for *BrAPK1* and *BrSUR1* in ‘Nanane’ (Supplementary Figure S3).

### Gene ontology analysis of differentially expressed genes following inoculation of *A. candida*

**Figure 3:**
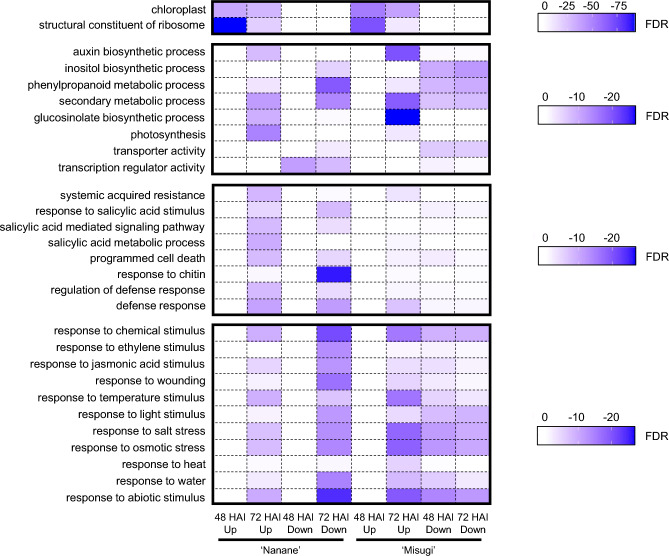
GO terms overrepresented in up and downregulated genes following *A. candida* (WMB01) inoculation. Log_10_ values of FDR are shown with the scale from white to purple. *HAI* hours after *A. candida* inoculation.

The up and downregulated genes at 48 or 72 HAI in the resistant or susceptible cultivar were categorized into GO cellular component (CC), GO molecular function (MF), and GO biological process (BP). 80 and 87 categories were overrepresented in upregulated DEGs at 48 HAI in ‘Nanane’ and ‘Misugi’, respectively (FDR < 0.001), and 71 categories overlapped (Supplementary Figure S4, Supplementary Tables S3, S4). Genes categorized into ‘structural constituent of ribosome’ and ‘chloroplast’ in CC were overrepresented in both cultivars (Fig. 3, Supplementary Tables S3, S4). Five and 103 categories were overrepresented in downregulated DEGs at 48 HAI in ‘Nanane’ and ‘Misugi’, respectively (FDR < 0.001), and one category overlapped (Supplementary Figure S4, Supplementary Tables S5, S6). In ‘Misugi’, stress response or stimulus-related terms and secondary metabolic process-related terms were overrepresented. The genes categorized into ‘transcription regulator activity’ in MF tended to be overrepresented in the downregulated genes at 48 HAI of ‘Nanane’ (Fig. 3, Supplementary Tables S5, S6).

379 and 367 categories were overrepresented in upregulated DEGs at 72 HAI in ‘Nanane’ and ‘Misugi’, respectively (FDR < 0.001), and 290 categories overlapped (Supplementary Figure S4, Supplementary Tables S7, S8). In ‘Misugi’, stress response or stimulus-related terms and secondary metabolic process-related terms tended to be overrepresented (Fig. 3, Supplementary Tables S7, S8). Defense response-related terms such as ‘systemic acquired resistance’, ‘programmed cell death’, and ‘regulation of defense response’, SA-related terms such as ‘salicylic acid metabolic process’ and ‘salicylic acid mediated signaling pathway’, and photosynthesis-related terms tended to be overrepresented in ‘Nanane’ (Fig. 3, Supplementary Tables S7, S8). 201 and 116 categories were overrepresented in downregulated DEGs at 72 HAI in ‘Nanane’ and ‘Misugi’, respectively (FDR < 0.001), of which 58 categories overlapped (Supplementary Figure S4, Supplementary Tables S9, S10). Genes categorized into ‘secondary metabolic process’ in BP were overrepresented in both cultivars. In ‘Misugi’, genes categorized into ‘transporter activity’ in MF were overrepresented (Fig. 3, Supplementary Tables S9, S10). The genes categorized into ‘transcription regulator activity’ in MF were overrepresented and stress response or stimulus-related terms tended to be overrepresented in ‘Nanane’ (Fig. 3, Supplementary Tables S9, S10).

### Comparison between differentially expressed genes by *A. candida* inoculation and SA-responsive genes

**Figure 4:**
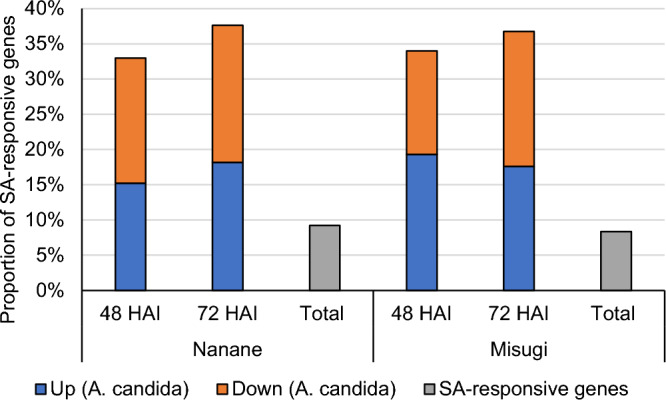
Proportion of salicylic acid (SA)-responsive genes whose expression was up or downregulated by *A. candida* (WMB01) inoculation. “Total” represents the proportion of SA-responsive genes in all genes. *HAI* hours after *A. candida* inoculation.

We identified 3780 and 3423 SA-responsive genes in ‘Nanane’ and ‘Misugi’, respectively^37^. Genes categorized into ‘salicylic acid mediated signaling pathway’ were overrepresented in up and downregulated genes at 72 HAI following *A. candida* inoculation in ‘Nanane’ (Fig. 3). It was predicted that some SA-responsive genes would overlap with DEGs following *A. candida* inoculation. Of 3780 SA-responsive genes in ‘Nanane’, 572 of 1735 (33.0%) DEGs at 48 HAI and 549 of 1459 (37.6%) DEGs at 72 HAI overlapped (Fig. 4, Table 1). Of 3423 SA-responsive genes in ‘Misugi’, 463 of 1363 (34.0%) DEGs at 48 HAI and 518 of 1409 (36.8%) DEGs at 72 HAI overlapped (Fig. 4, Table 1). These results showed that in both cultivars, DEGs following *A. candida* inoculation accounted for a high proportion of SA-responsive genes at both 48 and 72 HAI.

Of 59 genes that showed upregulation by SA treatment in both *B. rapa* and *A. thaliana*^37^, 12 and nine genes were upregulated at 48 and 72 HAI in ‘Nanane’, and eight genes were common between two time points, including *DOWNY MILDEW RESISTANT 6* (*DMR6*) and *WRKY DNA-BINDING PROTEIN 54* (*WRKY54*) (Supplementary Table S11). Only a few genes were upregulated at 48 HAI or 72 HAI in ‘Misugi’ (Supplementary Table S11).

**Figure 5:**
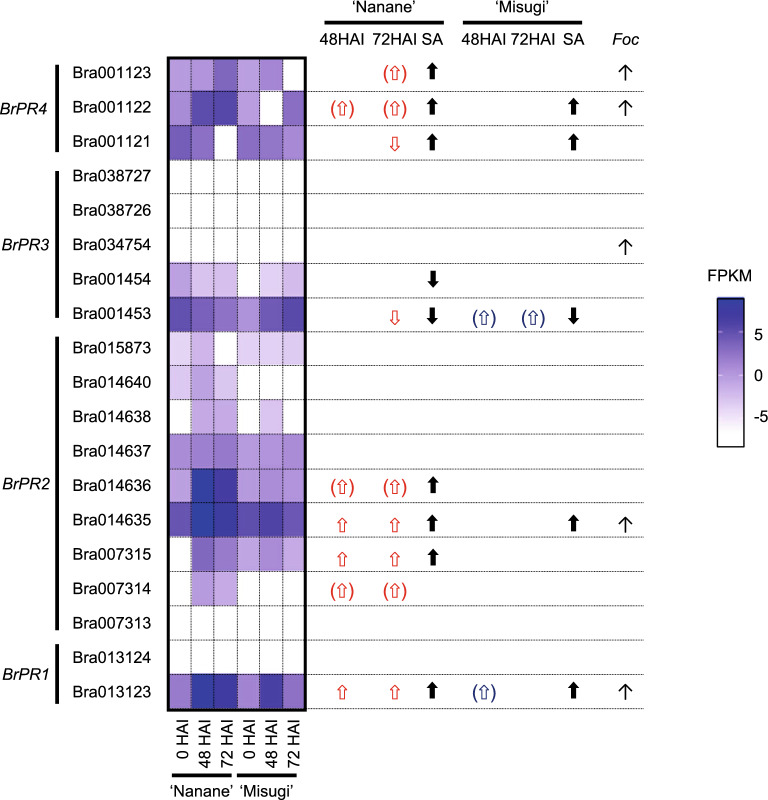
Expression pattern of 19 *BrPR* genes following *A. candida* (WMB01) inoculation. Log_2_ values of fragments per kilobase per million (FRKM) are shown with the scale from white to purple. Parentheses indicate the upregulation but not significantly. “SA” represents up or downregulated at 72 h after salicylic acid (SA) treatments in ‘Nanane’ or ‘Misugi’^37^. “*Foc*” represents upregulated after 24 h *Fusarium oxysporum* f. sp. *conglutinans* (*Foc*) inoculation in a resistant line, RJKB-T23^34^. *HAI* hours after *A. candida* inoculation.

Of 19 *BrPR* genes identified previously^37^, one of two *BrPR1* genes and two of nine *BrPR2* genes were upregulated at both 48 and 72 HAI in ‘Nanane’, and one of three *BrPR4* genes was downregulated at 72 HAI in ‘Nanane’. One of the five *BrPR3* genes was downregulated at 72 HAI in ‘Nanane' (Fig. 5). In ‘Misugi’, none of the 19 *BrPR* genes showed any change in expression levels (Fig. 5). Two *BrPR2* and two *BrPR4* in ‘Nanane’ and one *BrPR3* gene in ‘Misugi’ tended to be upregulated, although they were not identified as DEGs in the RNA-seq analysis (Fig. 5).

Genes categorized into ‘systemic acquired resistance’ also tended to show differential expression following *A. candida* inoculation in ‘Nanane’ but not in ‘Misugi’ (Supplementary Table S12).

### Comparison between differentially expressed genes following *A. candida* and *Fusarium oxysporum* inoculation

**Figure 6:**
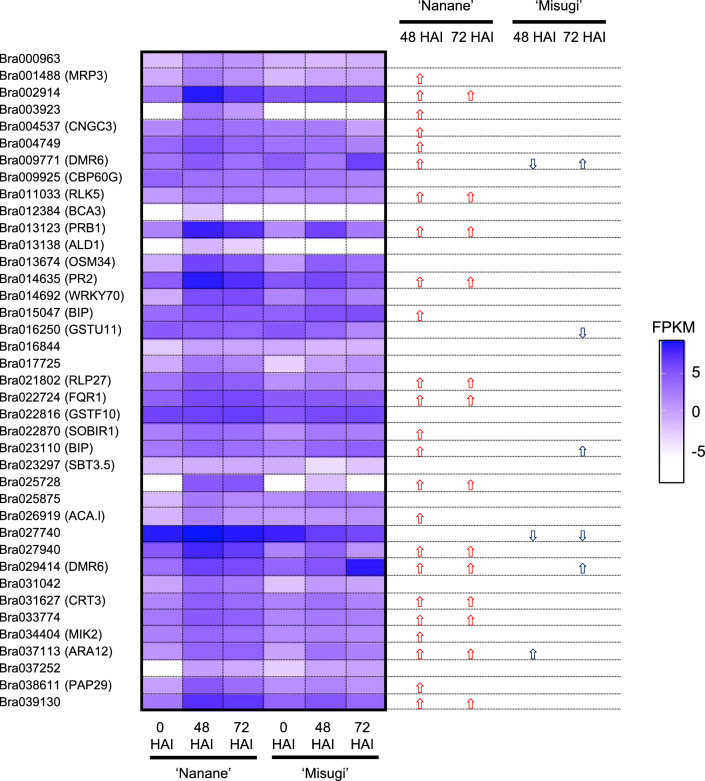
Expression pattern following *A. candida* (WMB01) inoculation in 39 salicylic acid (SA)-induced genes specific to Fusarium yellows resistant line. Log_2_ values of fragments per kilobase per million (FRKM) are shown with scale from white to purple. *HAI* hours after *A. candida* inoculation.

Previously, we performed RNA-seq using *B. rapa* Fusarium yellows resistant and susceptible lines with and without *F. oxysporum* f. sp. *conglutinans* (*Foc*) inoculation at 24 and 72 HAI and identified DEGs between inoculated and non-inoculated samples (DRA005538 and DRA005976)^34^. Gene ontology analysis using upregulated genes in inoculated samples at 24 HAI showed an overrepresentation of the category, systemic acquired resistance, in resistant line^34^. Later, we identified upregulated genes following *Foc* inoculation that overlapped with SA-induced genes^37^. From these studies, we suggested that the defense response to *Foc* is mediated by SA-induced genes at 24 HAI in *B. rapa*^34,37^. Of 245 upregulated genes after *Foc* inoculation in the Fusarium yellows resistant line (RJKB-T23) in *B. rapa*, 47 (19%) and 25 genes (10%) were also upregulated after *A. candida* inoculation at 48 and 72 HAI in ‘Nanane’, and 19 genes overlapped between two time points (Table 1). In ‘Misugi’, 18 (7%) and 26 genes (11%) were also upregulated after *A. candida* inoculation at 48 and 72 HAI, and 16 genes overlapped between two time points (Table 1). However, between cultivars at the same time points, only a few upregulated genes overlapped (one at 48 HAI and four at 72 HAI) (Table 1). Some SAR-related genes and *BrPR* genes overlapped between upregulated genes in both Fusarium yellows resistant line and white rust resistant cultivar (Fig. 5, Supplementary Table S12), while no gene overlapped between upregulated genes in both Fusarium yellows resistant line and white rust susceptible cultivar (Fig. 5, Supplementary Table S12). Previously, we identified 39 SA-induced genes specific to Fusarium yellows resistant line^37^. Of these 39 genes, 24 (61.5%) and 13 (33.3%) genes were upregulated by *A. candida* inoculation at 48 and 72 HAI, respectively, in ‘Nanane’, while a few genes were upregulated at 48 and 72 HAI in ‘Misugi’ (Fig. 6), suggesting a similar defense response mediated by SA occurred in resistant cultivar/line between two different pathogens, especially at 48 HAI by *A. candida* inoculation and 24 HAI with *Foc* inoculation.

## Discussion

In this study, we used two *A. candida* isolates; WMB01 was isolated from Mibuna and WKB01 was isolated from a turnip, in a different year and in a different field. Pathogenicity was confirmed by repeated inoculation tests in the controlled condition (using a growth chamber), and it was not changed by seasons or repeated inoculation. Because *A. candida* has some host specificity^46^, we compared the pathogenicity of WMB01 and WKB01 to *B. rapa*, and there was no difference in pathogenicity between the two isolates, suggesting that WMB01 and WKB01 are the same race. In cotyledons, all six genes showed similar expression patterns between WMB01 and WKB01 inoculation at 72 HAI. We also compared the expression pattern following WKB01 inoculation between cotyledons and true leaves, and most genes showed similar expression patterns between these two tissues. These results suggest that our RNA-seq data using single tissue and single isolate could reflect the transcriptional change following *A. candida* inoculation. However, race or tissue-specific transcriptional change following *A. candida* inoculation could be included in our RNA-seq data, and RNA-seq data using multiple isolates and tissues will be able to exclude these effects.

We identified DEGs at 48 and 72 HAI following *A. candida* inoculation. About 3–4% of total genes showed changed gene expression following *A. candida* inoculation in the white rust resistant (‘Nanane’) and susceptible (‘Misugi’) cultivars. Nearly half of the DEGs (40–55%) overlapped between the resistant and susceptible cultivars at 48 and 72 HAI. Most overrepresented GO terms in upregulated genes overlapped between the resistant and susceptible cultivars at 48 HAI, and three-quarters of overrepresented GO terms in upregulated genes at 72 HAI overlapped between them. In contrast, overrepresented GO terms in downregulated genes at 48 and 72 HAI were cultivar specific; more than 100 cultivar specific GO terms were identified in ‘Misugi’ at 48 HAI and in ‘Nanane’ at 72 HAI. These results suggest that the function of transcriptionally responsive genes following *A. candida* inoculation differs between the resistant and susceptible cultivars at 48 and 72 HAI, particularly for downregulated genes. Similar differences in the function of transcriptional responsive genes between the resistant and susceptible inbred lines have been observed in *Foc* inoculation^34^. In the case of *Foc* inoculation, 50% of the upregulated genes at 24 HAI overlapped between resistant and susceptible inbred lines, but at 72 HAI only 10% overlapped making the difference between the resistant and susceptible lines clear. The percentage of overlapped downregulated genes between resistant and susceptible inbred lines was low at both 24 and 72 HAI^34^.

In the *A. candida* response, some overrepresented GO terms were cultivar specific at 48 or 72 HAI. At 48 HAI, a small number of cultivar-specific GO terms were present in upregulated genes. Genes involved in the GO terms related to abiotic stress response or ‘secondary metabolic process’ tended to be downregulated at 48 HAI in the susceptible cultivar, while these GO terms were overrepresented in upregulated and downregulated genes at 72 HAI in both resistant and susceptible cultivars. This difference might be due to the different degrees of pathogen infection between resistant and susceptible cultivars at 48 HAI. At 72 HAI, the expression of genes categorized into SA response-related terms or ‘program cell death’ tended to be up and downregulated in the resistant cultivar. The genes categorized into ‘systemic acquired resistance’ tended to be upregulated in the resistant cultivar. These results also reflect different degrees of pathogen infection and suggest that hypersensitive reactions could occur in the resistant cultivar. The expression of genes categorized into ‘auxin biosynthetic process’ tended to be upregulated in the susceptible cultivar. Upregulation of genes categorized into ‘auxin biosynthetic process’ by *Foc* inoculation was also observed in the susceptible inbred line^34^. The pathogens manipulate plant auxin biosynthesis and signaling that result in susceptibility of host plants; SA induced by biotrophic pathogen infection can reduce the biosynthesis of auxin to lessen susceptibility^47^. The maize pathogen effector of *Ustilago maydis* prevents transcriptional of a repressor of auxin signaling to upregulate auxin signaling and promote susceptibility^48^. Upregulation of genes categorized into ‘auxin biosynthetic process’ by *A. candida* or *Foc* inoculation in the susceptible cultivar/inbred line is common, and ETI could suppress the upregulation of genes involved in ‘auxin biosynthetic process’ in the resistant cultivar/inbred line.

SA plays an essential role in defense response, and genes categorized into SA response-related terms were overrepresented in upregulated genes at 72 HAI in the resistant cultivar. We identified SA-responsive genes in ‘Nanane’ and ‘Misugi’^37^, and 30–40% of DEGs overlapped with SA-responsive genes in both cultivars. These percentages are higher than the percentages of SA-responsive genes in all genes (8–9%), and there was no difference in the percentages of SA-responsive genes in DEGs between resistant and susceptible cultivars, suggesting that expression of SA-responsive genes tended to be affected by *A. candida* inoculation in both cultivars. About 34% of DEGs overlapped with SA-responsive genes in both resistant and susceptible cultivars, these percentages were lower than the percentages of SA-responsive genes that were common in both cultivars (54–60%), suggesting that SA-responsive genes with different roles in resistant and susceptible cultivars were changed in expression by *A. candida* inoculation. For *PR* genes and SAR-related genes, which are important for the defense response and are SA-responsive, the number of DEGs is higher in the resistant cultivar than in the susceptible cultivar. Of SA-responsive genes conserved in *A. thaliana* and *B. rapa* that might have an important function in Brassicaceae, the number of DEGs is also higher in the resistant cultivar than in the susceptible cultivar. More genes related to the disease response may be identified as DEGs, which respond to SA in the resistant cultivar than in the susceptible cultivar.

Previous studies suggest that SA-dependent SAR plays a role in defense response in the hemibiotrophic pathogen, *Foc*, and 39 candidate genes that might function in defense response to *Foc* were identified^34,37^. More of these 39 genes were identified as DEGs following *A. candida* inoculation in the resistant cultivar than in the susceptible cultivar, with 60% of DEGs overlapping at 48 HAI in the resistant cultivar. These results suggest that there may be some commonality in the defense response against the two different types of pathogens, *A. candida* and *Foc*. *BrPR2*, *BASIC PATHOGENESIS-RELATED PROTEIN 1* (*BrPRB1*), *BrDMR6*, *LUMINAL BINDING PROTEIN*, *SUPPRESSOR OF BIR1 1* (*BrSOBIR1*), and *MDIS1-INTERACTING RECEPTOR LIKE KINASE2* (*BrMIK2*) were upregulated by both *A. candida* and *Foc* inoculation in the resistant cultivar/inbred line. DMR6 belongs to the superfamily of 2-oxoglutarate Fe (II)-dependent dioxygenases and is known as a susceptibility gene in *A. thaliana*^49,50^. Inactivation of *DMR6* leads to resistance to different types of pathogens in *A. thaliana*^51^. Loss of function of a *DMR6* ortholog in tomato and potato also showed disease resistance against different classes of pathogens^52,53^. *BrDMR6* genes were upregulated by *Foc* inoculation in both resistant and susceptible inbred lines, and coordinate upregulation of one *BrDMR6* gene and its paired natural antisense transcripts in the resistant inbred line was observed^34,54^. Following *A. candida* inoculation, *BrDMR6* genes were upregulated at 48 HAI in the resistant cultivar and then decreased at 72 HAI to a level, which was still higher than in non-inoculated plants. *BrDMR6* genes were upregulated at 72 HAI in the susceptible cultivar, and the expression level of *BrDMR6* was eight times higher than in the resistant cultivar. These results suggest that BrDMR6 could be involved in susceptibility to *A. candida* in the susceptible cultivar and the defense response to *A. candida* inoculation in the resistant cultivar could repress *BrDMR6* expression. The leucine-rich repeat receptor protein, *SOBIR1*, which plays a role in PTI in *A. thaliana*^55^, was upregulated by *A. candida* or *Foc* inoculation in the resistant cultivar/inbred line, suggesting that *BrSOBIR1* is involved in defense response against *A. candida* and *Foc*. In *A. thaliana*, the leucine-rich repeat receptor-like kinase MIK2 is involved in resistance to *F. oxysporum* and may recognize elicitors of *F. oxysporum* to induce PTI^56–58^. Although the *mik2* mutant in *A. thaliana* showed susceptibility to *F. oxysporum*, it did not show susceptibility to leaf pathogens, suggesting that MIK2 is involved in resistance to the specific pathogen in the root^56^. However, *BrMIK2* was upregulated by both *A. candida* and *Foc* inoculation in the resistant cultivar/inbred line of *B. rapa*, suggesting that *BrMIK2* might be involved in deference response against these two pathogens.

Comparison among DEGs of *A. candida* inoculation, SA treatment, and *Foc* inoculation identified candidate genes involved in the defense response to *A. candida* in *B. rapa*. Transcriptome analysis following *A. candida* inoculation in the resistant and susceptible cultivars showed that activation of genes involved in defense responses such as SAR and programmed cell death was observed in the resistant cultivar. Similarly, transcriptome analysis following *Foc* inoculation in the resistant and susceptible inbred lines showed that activation of genes involved in defense responses such as SAR at 24 HAI was observed only in the resistant inbred line. *F. oxysporum* is a hemibiotrophic pathogen, and its infection cycle starts as a biotrophic phase and changes to a necrotrophic phase^59^. Our previous transcriptome suggested that *Foc* changes their phase from biotrophy to necrotrophy in susceptible line from 24 to 72 HAI^34,37^. SAR plays a role in the defense response to biotrophic (*A. candida*) and biotrophic phase of hemibiotrophic (*Foc*) pathogens, and common SA-responsive genes were upregulated following *A. candida* and *Foc* inoculation in the resistant cultivar/inbred line. In the resistant cultivar/inbred line, resistance to these two pathogens is mediated by the immediate induction of SAR via biosynthesis of salicylic acid, which could be induced by the R protein recognizing AVR; the defense pathway downstream of ETI is shared between *A. candida* and *Foc*. Twenty-four SA-induced genes, which were upregulated by *A. candida* and *Foc* inoculation in resistant cultivar/inbred line, including *BrPR2*, *BrPRB1*, *BrDMR6*, *BrSOBIR1*, and *BrMIK2*, are candidates for the defense response through SA by *A. candida* and *Foc* infection in *B. rapa*. This study will provide important data for future understanding of resistance mechanisms against *A. candida* in *B. rapa*.

In this study, we performed RNA-seq in a single-stage under-regulated growth condition. However, the actual growing environment in the field is more complex. To examine whether our results can apply to a real growing environment field condition, RNA-seq analysis in the field will be important and will enable us to understand the transcript overview of *B. rapa* by *A. candida* inoculation.

## Supplementary Information

Supplementary Figures.

Supplementary Tables.

## Data Availability

The sequence data have been submitted to the DDBJ database (http://www.ddbj.nig.ac.jp) under accession numbers DRA013255 and DRA014486.

## References

[1] Cheng F (2016). Subgenome parallel selection is associated with morphotype diversification and convergent crop domestication in *Brassica rapa* and *Brassica oleracea*. Nat. Genet..

[2] Neik TX, Barbetti MJ, Batley J (2017). Current status and challenges in identifying disease resistance genes in *Brassica napus*. Front. Plant Sci..

[3] Lv H, Kole C (2020). The importance of genetic and epigenetic research in the *Brassica* vegetables in the face of climate change. Genomic Designing of Climate-Smart Vegetable Crops.

[4] Zhang K (2021). Challenges and prospects for a potential allohexaploid *Brassica* crop. Theor. Appl. Genet..

[5] UN (1935). Genome analysis in Brassica with special reference to the experimental formation of *B. napus* and peculiar mode of fertilization. Jpn. J. Bot..

[6] Fujimoto R (2018). Recent research on the mechanism of heterosis is important for crop and vegetable breeding systems. Breed. Sci..

[7] Lv H, Fang Z, Yang L, Zhang Y, Wang Y (2020). An update on the arsenal: Mining resistance genes for disease management of *Brassica* crops in the genomic era. Hortic. Res..

[8] Mehraj H (2020). Genetics of clubroot and Fusarium wilt disease resistance in Brassica vegetables: The application of marker assisted breeding for disease resistance. Plants.

[9] Saharan G, Verma P, Meena P, Kumar A (2014). The Disease in White Rust of Crucifers: Biology, Ecology and Management.

[10] Meena PD, Verma PR, Saharan GS, Borhan MH (2014). Historical perspectives of white rust caused by *Albugo candida* in oilseed Brassica. J. Oilseed Brass..

[11] Singh KP, Kumari P, Rai PK (2021). Current status of the disease-resistant gene(s)/QTLs, and strategies for improvement in *Brassica juncea*. Front. Plant Sci..

[12] Jones JDG, Dangl JL (2006). The plant immune system. Nature.

[13] Dodds PN, Rathjen JP (2010). Plant immunity: Towards an integrated view of plant–pathogen interactions. Nat. Rev. Genet..

[14] Yuan M (2021). Pattern-recognition receptors are required for NLR-mediated plant immunity. Nature.

[15] Yuan M, Ngou BPM, Ding P, Xin XF (2021). PTI-ETI crosstalk: An integrative view of plant immunity. Curr. Opin. Plant Biol..

[16] Lu Y, Tsuda K (2021). Intimate association of PRR- and NLR-mediated signaling in plant immunity. Mol. Plant Microbe Interact..

[17] Glazebrook J (2005). Contrasting mechanisms of defense against biotrophic and necrotrophic pathogens. Annu. Rev. Phytopathol..

[18] Bari R, Jones JDG (2009). Role of plant hormones in plant defence responses. Plant Mol. Biol..

[19] Pieterse CMJ, Van der Does D, Zamioudis C, Leon-Reyes A, Van Wees SCM (2012). Hormonal modulation of plant immunity. Annu. Rev. Cell Dev. Biol..

[20] Klessig DF, Choi HW, Dempsey DA (2018). Systemic acquired resistance and salicylic acid: Past, present, and future. Mol. Plant Microbe Interact..

[21] Caarls L, Pieterse CM, Van Wees SCM (2015). How salicylic acid takes transcriptional control over jasmonic acid signaling. Front. Plant Sci..

[22] Shigenaga AM, Argueso CT (2016). No hormone to rule them all: Interactions of plant hormones during the responses of plants to pathogens. Semin. Cell Dev. Biol..

[23] Shigenaga AM, Berens ML, Tsuda K, Argueso CT (2017). Towards engineering of hormonal crosstalk in plant immunity. Curr. Opin. Plant Biol..

[24] Zhang W (2018). Different pathogen defense strategies in *Arabidopsis*: More than pathogen recognition. Cells.

[25] Garber M, Grabherr MG, Guttman M, Trapnell C (2011). Computational methods for transcriptome annotation and quantification using RNA-seq. Nat. Methods.

[26] Saeki N (2016). Molecular and cellular characteristics of hybrid vigour in a commercial hybrid of Chinese cabbage. BMC Plant Biol..

[27] Shea DJ (2019). Long noncoding RNAs in *Brassica rapa* L. following vernalization. Sci. Rep..

[28] Stark R, Grzelak M, Hadfield J (2019). RNA sequencing: The teenage years. Nat. Rev. Genet..

[29] Wang NN (2021). Phosphorylation of WRKY16 by MPK3-1 is essential for its transcriptional activity during fiber initiation and elongation in cotton (*Gossypium hirsutum*). Plant Cell.

[30] Neik TX, Amas J, Barbetti M, Edwards D, Batley J (2020). Understanding host–pathogen interactions in *Brassica napus* in the omics era. Plants.

[31] Campos MD, Félix MDR, Patanita M, Materatski P, Varanda C (2021). High throughput sequencing unravels tomato-pathogen interactions towards a sustainable plant breeding. Hortic. Res..

[32] Chen J, Pang W, Chen B, Zhang C, Piao Z (2016). Transcriptome analysis of *Brassica rapa* near-isogenic lines carrying clubroot-resistant and -susceptible alleles in response to *Plasmodiophora brassicae* during early infection. Front. Plant Sci..

[33] Yuan Y (2021). Transcriptome and coexpression network analyses reveal hub genes in Chinese cabbage (*Brassica rapa* L. ssp. pekinensis) during different stages of *Plasmodiophora brassicae* infection. Front. Plant Sci..

[34] Miyaji N (2017). Comparison of transcriptome profiles by *Fusarium oxysporum* inoculation between Fusarium yellows resistant and susceptible lines in *Brassica rapa* L.. Plant Cell Rep..

[35] Liu M (2019). Comparative transcriptome analysis reveals defense responses against soft rot in Chinese cabbage. Hortic. Res..

[36] Zheng H (2020). Comparative transcriptome analysis between a resistant and a susceptible Chinese cabbage in response to *Hyaloperonospora brassicae*. Plant Signal Behav..

[37] Miyaji N, Shimizu M, Takasaki-Yasuda T, Dennis ES, Fujimoto R (2021). The transcriptional response to salicylic acid plays a role in Fusarium yellows resistance in *Brassica rapa* L.. Plant Cell Rep..

[38] Bolger AM, Lohse M, Usadel B (2014). Trimmomatic: A flexible trimmer for Illumina sequence data. Bioinformatics.

[39] Kim D, Paggi JM, Park C, Bennett C, Salzberg SL (2019). Graph-based genome alignment and genotyping with HISAT2 and HISAT-genotype. Nat. Biotechnol..

[40] Trapnell C (2012). Differential gene and transcript expression analysis of RNA-seq experiments with TopHat and Cufflinks. Nat. Protoc..

[41] Du Z, Zhou X, Ling Y, Zhang Z, Su Z (2010). agriGO: A GO analysis toolkit for the agricultural community. Nucleic Acids Res..

[42] Shimizu M (2014). Identification of candidate genes for fusarium yellows resistance in Chinese cabbage by differential expression analysis. Plant Mol. Biol..

[43] Akter A (2020). Gene expression analysis in response to vernalization in Chinese cabbage (*Brassica rapa* L.). Hortic. J..

[44] Miyaji N (2021). Development of a new DNA marker for Fusarium yellows resistance in *Brassica rapa* vegetables. Plants.

[45] Fujimoto R, Sasaki T, Nishio T (2006). Characterization of DNA methyltransferase genes in *Brassica rapa*. Genes. Genet. Syst..

[46] Petkowski JE, Minchinton E, Thomson F, Faggian R, Cahill D (2010). Races of *Albugo candida* causing white blister rust on *Brassica* vegetables in Australia. Acta Hortic..

[47] Zhong Q, Hu H, Fan B, Zhu C, Chen Z (2021). Biosynthesis and roles of salicylic acid in balancing stress response and growth in plants. Int. J. Mol. Sci..

[48] Navarrete F (2022). TOPLESS promotes plant immunity by repressing auxin signaling and is targeted by the fungal effector Naked1. Plant Commun..

[49] van Damme M (2005). Identification of *Arabidopsis* loci required for susceptibility to the downy mildew pathogen *Hyaloperonospora parasitica*. Mol. Plant Microbe Interact..

[50] van Damme M, Huibers RP, Elberse J, Van den Ackerveken G (2008). Arabidopsis *DMR6* encodes a putative 2OG-Fe(II) oxygenase that is defense-associated but required for susceptibility to downy mildew. Plant J..

[51] Zeilmaker T (2015). DOWNY MILDEW RESISTANT 6 and DMR6-LIKE OXYGENASE 1 are partially redundant but distinct suppressors of immunity in Arabidopsis. Plant J..

[52] Kieu NP, Lenman M, Wang ES, Petersen BL, Andreasson E (2021). Mutations introduced in susceptibility genes through CRISPR/Cas9 genome editing confer increased late blight resistance in potatoes. Sci. Rep..

[53] Thomazella DPT (2021). Loss of function of a DMR6 ortholog in tomato confers broad-spectrum disease resistance. Proc. Natl. Acad. Sci. USA.

[54] Akter MA (2022). Transcriptional association between mRNAs and their paired natural antisense transcripts following *Fusarium oxysporum* inoculation in *Brassica rapa* L.. Horticulturae.

[55] Pruitt RN (2021). The EDS1-PAD4-ADR1 node mediates *Arabidopsis* pattern-triggered immunity. Nature.

[56] Van der Does D (2017). The Arabidopsis leucine-rich repeat receptor kinase MIK2/LRR-KISS connects cell wall integrity sensing, root growth and response to abiotic and biotic stresses. PLoS Genet..

[57] Coleman AD (2021). The Arabidopsis leucine-rich repeat receptor-like kinase MIK2 is a crucial component of early immune responses to a fungal-derived elicitor. New Phytol..

[58] Rhodes J (2021). Perception of a divergent family of phytocytokines by the Arabidopsis receptor kinase MIK2. Nat. Commun..

[59] Lyons R (2015). *Fusarium oxysporum* triggers tissue-specific transcriptional reprogramming in *Arabidopsis thaliana*. PLoS One.

