# Supplementary material for: Differences in the transcriptional immune response to Albugo candida between white rust resistant and susceptible cultivars in Brassica rapa L
Source: Sci Rep. 2023 May 26;13:8599. doi: 10.1038/s41598-023-35205-5 (PMC10219939; doi:10.1038/s41598-023-35205-5)
Supplement: Supplementary file 1 — Supplementary Figures. [file 41598_2023_35205_MOESM1_ESM.pdf]

Title: Differences in the transcriptional immune response to *Albugo candida* between white rust resistant and susceptible cultivars in *Brassica rapa* L.

Naomi Miyaji[1,2,†], Mst. Arjina Akter[1,3,†], Motoki Shimizu[2], Hasan Mehraj[1], Md Asad-Ud Doullah[4], Elizabeth S. Dennis[5,6], Izumi Chuma[7], Ryo Fujimoto[1,*]

[1] Graduate School of Agricultural Science, Kobe University, Kobe 657-8501, Japan

[2] Iwate Biotechnology Research Center, Narita, Kitakami, Iwate 024-0003, Japan

[3] Department of Plant Pathology, Faculty of Agriculture, Bangladesh Agricultural University, Mymensingh-2202, Bangladesh

[4] Department of Plant Pathology and Seed Science, Faculty of Agriculture, Sylhet Agricultural University, Sylhet 3100, Bangladesh

[5] CSIRO Agriculture and Food, Canberra, ACT 2601, Australia

[6] School of Life Science, Faculty of Science, University of Technology Sydney, Broadway, NSW 2007, Australia.

[7] Obihiro University of Agriculture and Veterinary Medicine, Obihiro, 080-8555, Japan

† These authors have contributed equally to this work and share first authorship.

* Corresponding Author Ryo Fujimoto

Graduate School of Agricultural Science, Kobe University, Kobe 657-8501, Japan

TEL; +81-78-803-5827, FAX; +81-78-803-5829, e-mail; leo@people.kobe-u.ac.jp

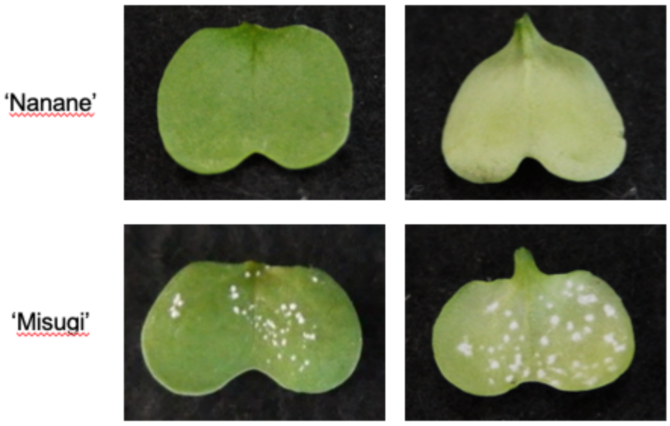

Fig. S1. Symptoms after *A. candida* inoculation in komatsuna cultivars 'Nanane' and 'Misugi'. Seven-day seedlings were inoculated by *A. candida* (WMB01). Cotyledons were harvested at 10 days after inoculation, and both sides of cotyledons (left, adaxial; right, abaxial) were photographed.

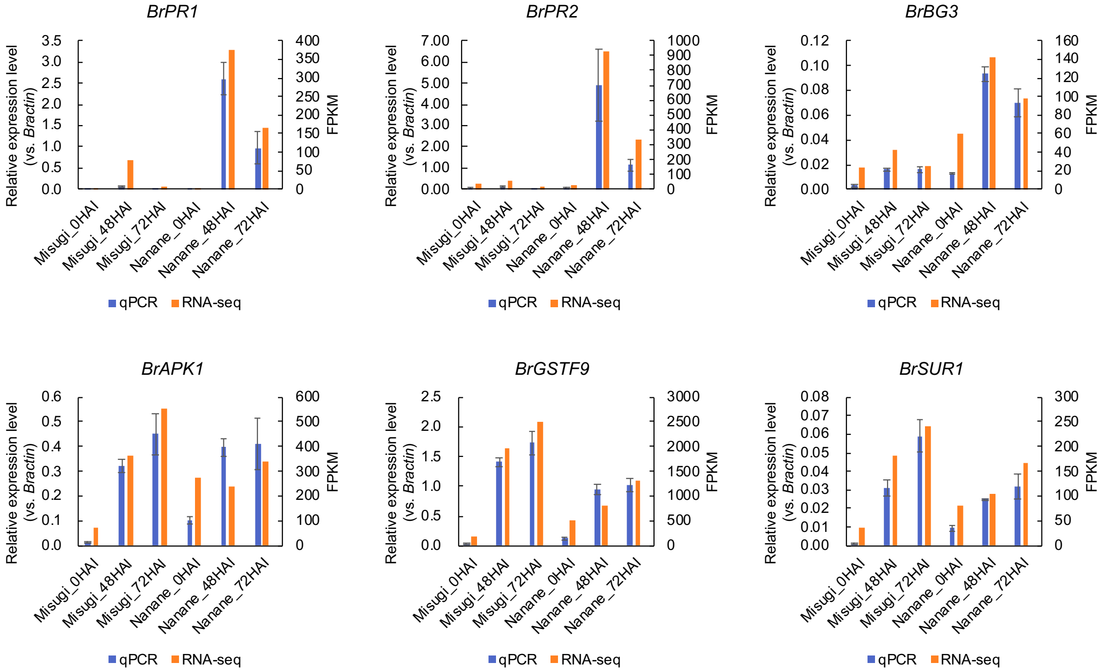

Fig. S2. Gene expression levels at 48 and 72 h after *A. candida* (WMB01) inoculation (HAI) measured by real-time RT-PCR (qPCR) and RNA-seq. By qPCR, the values are means ± SE (three biological and technical replicates) of relative expression levels compared with *Bractin*. The values of fragments per kilobase per million (FPKM) were calculated by data from RNA-seq analysis.

(a) Cotyledons

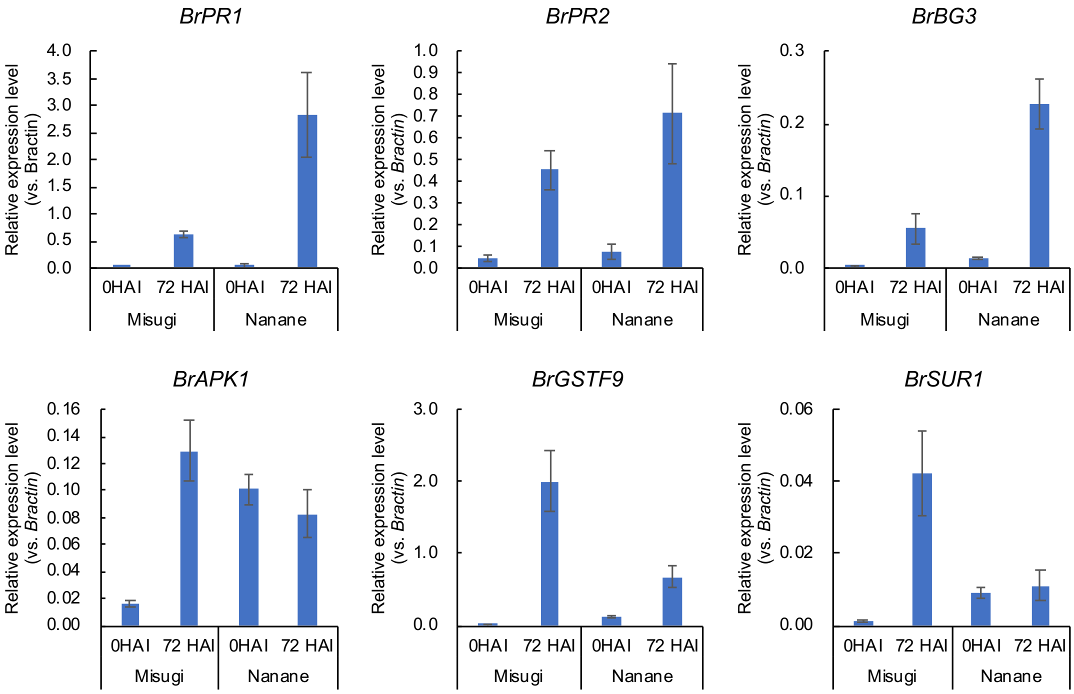

(b) True leaves

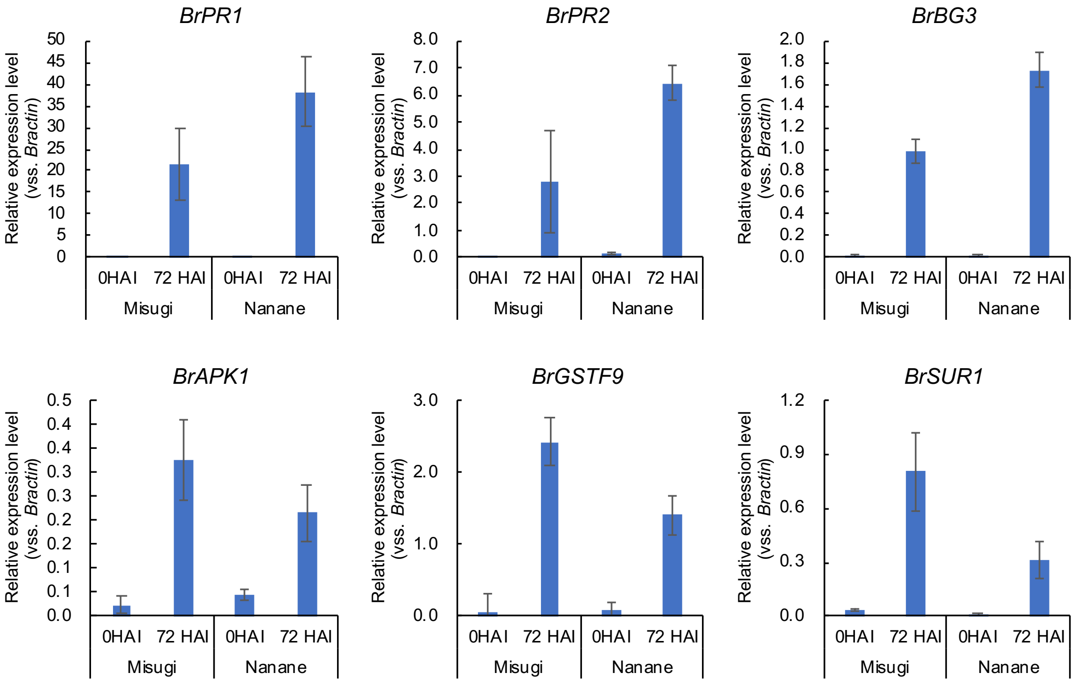

Fig. S3. Gene expression levels in cotyledons (a) and true leaves (b) at 72 h after *A. candida* (WKB01) inoculation (HAI) measured by real-time RT-PCR (qPCR). The values are means ± SE (three biological and technical replicates) of relative expression levels compared with *Bractin*.

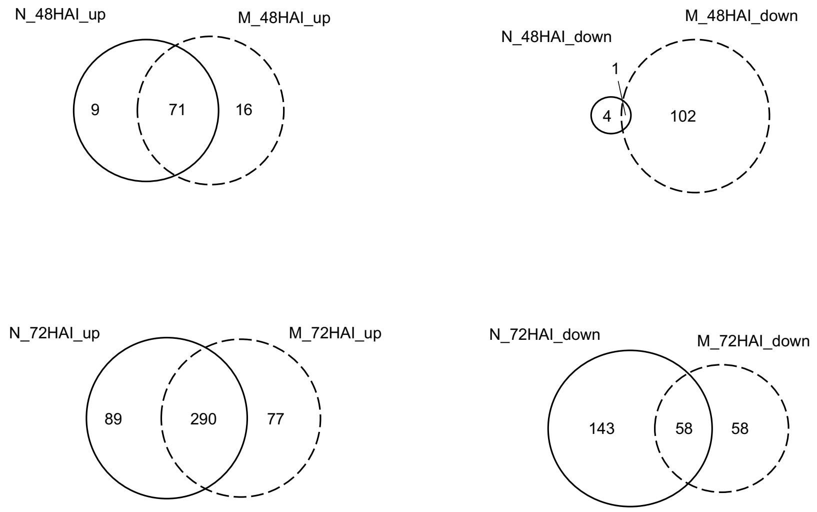

Fig. S4. Venn diagram showing number of GO terms overrepresented in up or downregulated genes following *A. candida* inoculation in 'Nanane' (N) and 'Misugi' (M). HAI, hours after *A. candida* inoculation.
